# Effectiveness of virtual reality as a behavioural management method in children during dental visits: a systematic review

**DOI:** 10.3389/froh.2026.1798462

**Published:** 2026-06-04

**Authors:** Rand Ra'ed-Naser, Marcela Arenas-González, María Biedma-Perea, María José Barra-Soto, Carolina Caleza-Jimenéz, David Ribas-Pérez

**Affiliations:** Department of Paediatric Dentistry, Faculty of Dentistry, University of Seville, Seville, Spain

**Keywords:** behaviour management, conduct management, dentistry, virtual reality, virtual reality therapy

## Abstract

**Importance:**

Behaviour management techniques converge on the fundamental aim of facilitating the child´s adaptation by promoting familiarity and gradual desensitisation, thereby reducing stress levels and enhancing cooperation during dental procedures.

**Objective:**

This study aimed to evaluate the use of virtual reality (VR) as a behavioural management technique to reduce anxiety in paediatric patients during dental treatments.

**Methods:**

A research strategy was conducted including articles that have been published in the past 5 years using MeSH Terms in five different databases (PubMed, Scopus, ScienceDirect, EMBASE, and Web of Science). The studies included in the review involved children under 17 years old who used virtual reality to control their anxiety in the dental clinic.

**Results:**

The use of VR in paediatric dentistry improved behaviour in children with higher positive responses in VR groups, vs. control groups. The Frankl scale showed significant improvements after the use of VR before treatment. Furthermore, audiovisual distraction with VR was the most effective technique for reducing fear and anxiety in uncooperative children.

**Interpretation:**

Virtual reality is an effective technique for managing behaviour and reducing anxiety in paediatric patients during dental treatments, often outperforming conventional techniques.

**Systematic Review Registration:**

https://www.crd.york.ac.uk/PROSPERO/view/CRD420251015748, PORSPERO CRD420251015748.

## 1 Introduction

Behaviour management techniques are fundamentally aimed at facilitating a child's adaptation to dental procedures by promoting familiarity and gradual desensitisation, thereby reducing stress levels and enhancing cooperation during dental procedures. This approach is crucial because dental anxiety is a significant concern in children, often leading to stress, lack of cooperation, and difficulty in completing necessary treatment. A negative initial dental experience can initiate a cycle of fear, potentially resulting in heightened anxiety or even dental phobia (1, 2). Therefore, these techniques strive to transform the child's perception of dental care from 'stressful' to a more positive and manageable experience.

Over the years, several basic methods have been employed to manage children's behaviour in the dental setting. These include the Tell-Show-Do (TSD) technique, which involves explaining, demonstrating, and then performing the procedure in an age-appropriate, non-threatening manner. Voice control, another established method which uses variations in the dentist's tone, volume, and rhythm of speech to guide the child’s behaviour. Positive reinforcement, which involves rewarding good behaviour through praise or small incentives, is also commonly used to help children associate dental visits with positive experiences (3, 4). These time-tested methods primarily rely on communication and behaviour shaping to lower anxiety and improve cooperation.

Modern technological advancements have significantly influenced behavioural management in paediatric dentistry, particularly through the introduction of immersive tools like virtual reality (VR) headsets. VR creates a simulated, immersive environment that captures a child’s attention and reduces their perception of anxiety during dental procedures. This technology provides innovative and engaging ways to manage anxiety and enhance the dental experience, extending beyond traditional distraction techniques like music, stories, or animated videos.

VR's ability to engage multiple senses and distract children from the dental environment contributes to its effectiveness. Numerous studies indicate that virtual reality is an effective technique for managing behaviour and reducing anxiety in paediatric patients during dental treatments, often outperforming conventional methods. Research has shown that VR can significantly reduce anxiety levels compared to traditional techniques (5, 6), with children using VR headsets reporting lower heart rates and less discomfort (7, 8). Audiovisual distraction with VR has been identified as the most effective technique for reducing fear and anxiety in uncooperative children. Furthermore, immersive VR has been found to yield the best outcomes in terms of anxiety reduction and behavioural improvement, followed by semi-immersive and non-immersive VR, and then the TSD method.

Despite the promising findings, the overall body of evidence supporting VR's effectiveness is currently weakened by a predominance of trials with a high risk of bias. Many studies have faced methodological limitations, including difficulties in blinding procedures, reliance on subjective behavioural outcome measures, and the absence of prospective trial registration or pre-specified analysis plans. The heterogeneity and imprecision across studies, which employed different behavioural and anxiety assessment tools and diverse VR modalities, further complicate the synthesis of findings. Therefore, there is a clear need for larger and more methodologically rigorous studies to strengthen the certainty of conclusions and provide more definitive insights into VR's effectiveness as a behavioural management tool in paediatric dentistry. This systematic review aims to evaluate the current research on VR's effectiveness, compare it to traditional methods, and identify the scales used for assessment, thereby contributing to a more robust understanding of its role in paediatric dental care.

## 2 Materials and methods

### 2.1 Protocol and registration

The review was registered in the PROSPERO database (the International Prospective Registry of Systematic Reviews hosted by the National Institute for Health Research, Centre for Reviews and Dissemination of the University of York. Registration Number: CRD420251015748.

### 2.2 Review question

1. Population: pediatric patients between 4 and 17 years with anxiety requiring treatment dental
2. Intervention: use of VRG as a behaviour management technique during treatment dental
3. Comparison: distraction techniques for managing anxiety in the patient pediatric
4. Outcome: values obtained through the application of the FRANKL scale to determine the decrease in anxiety after the use of VRG

In paediatric patients aged 4–17 years with anxiety requiring dental treatment, is the use of VRG as a behaviour management technique more effective than other distraction techniques in reducing anxiety, as measured by the Frankl scale?

### 2.3 Search strategy

A comprehensive search strategy was conducted to identify articles published within the last five years, focusing on paediatric-related terms across five databases: PubMed, Scopus, ScienceDirect, EMBASE, and Web of Science.

The following search terms were used (last updated: 13 October 2025): (“virtual reality therapy” OR “virtual reality exposure therapy” OR “VR therapy” OR “virtual reality”) AND (“anxiety” OR “dental anxiety” OR “stress”) AND (“conduct management” OR “behaviour management” OR “patient management” OR “pain management”) AND (“dentistry” OR “dental treatment” OR “paediatric dentistry”). Boolean operators AND and OR were used to combine search terms appropriately, ensuring the inclusion of all potentially relevant articles. Truncation and field tags were adjusted to fit the syntax requirements of each database.

A simplified version of the search string was also applied: (“virtual reality therapy” OR “virtual reality”) AND anxiety AND (“conduct management” OR “behaviour management”) AND dentistry. Reference lists of included articles and relevant reviews were also manually screened to identify additional eligible studies.

### 2.4 Selection of studies

The selection procedure was independently performed by two reviewers (R-N.R and R-P.D), with any discrepancies resolved through discussion until consensus was achieved.

### 2.5 Inclusion and exclusion criteria

The inclusion criteria were defined to ensure that only studies relevant to the research question were considered. Eligible studies included children aged 4–17 years, in which virtual reality goggles (VRG) were used as an intervention during dental treatment. Studies were required to compare VRG with other distraction techniques, such as television, mobile devices, or audio stimuli. Only studies that evaluated the effectiveness of behavior management using VRG to reduce anxiety in children were included. Eligible study designs comprised clinical trials, randomised clinical trials, and randomised controlled studies. Studies were excluded if they involved children under four years of age or patients with disabilities, or if the intervention was conducted in hospital settings rather than dental clinics.

Research using exclusively the tell–show–do (TSD) technique as the comparison method was also excluded. Furthermore, studies assessing first dental visits without comparison to subsequent procedures, or those that did not employ the Frankl Behaviour Rating Scale, were not included. Systematic reviews, meta-analyses, and pilot studies were excluded as well.

### 2.6 Data extraction

From each eligible study, the following information was systematically extracted and tabulated: author(s), year of publication, study design, dependent variable, study groups, main findings, and conclusions. Although a quantitative meta-analysis was originally intended, the substantial clinical and methodological heterogeneity across the included studies precluded performing it.

### 2.7 Assessment of risk of bias

The risk of bias in the included studies was evaluated using the revised Cochrane Risk of Bias tool for randomised trials (RoB 2) (9). Potential sources of bias were assessed across the following domains: randomisation process, deviations from intended interventions, missing outcome data, measurement of outcomes, and selection of the reported results. The evaluation was independently carried out by two reviewers (A-G.M and D.R-P.), and any discrepancies were resolved through deliberation until consensus was achieved, thereby ensuring methodological rigour and reliability.

### 2.8 Analysis of the GRADE levels of evidence

The certainty of the evidence derived from the randomized controlled trials and *in vivo* studies was appraised independently using the GRADE approach (Grading of Recommendations, Assessment, Development, and Evaluation) (10). This framework provides a structured method for evaluating the overall quality of evidence by considering critical domains such as risk of bias, inconsistency, indirectness, imprecision, and potential publication bias. Data from the included studies were synthesized narratively due to clinical and methodological heterogeneity. Based on this assessment, the quality of evidence regarding behavioural management was categorised as high, moderate, low, or very low, with concise explanations provided to support each rating.

## 3 Results

**Figure 1:**
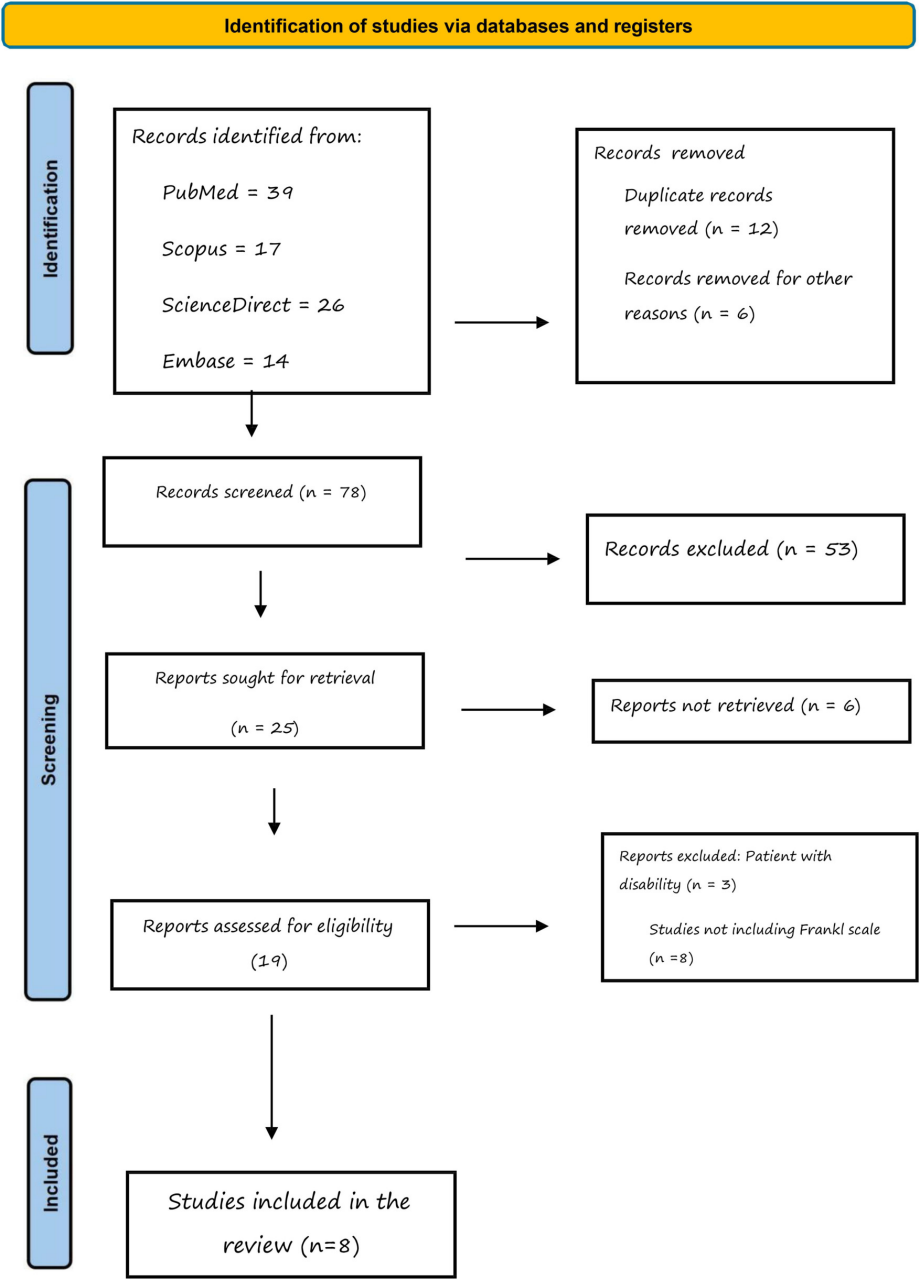
PRISMA 2020 flow diagram showing the study selection process.

The study selection process followed the PRISMA 2020 guidelines (11). A total of 96 records were identified from PubMed, Scopus, ScienceDirect, Embase, and Web of Science. After removing 18 duplicates or ineligible records, 78 articles were screened, resulting in the exclusion of 53. Of the 25 reports sought for retrieval, 6 could not be obtained. 19 studies were assessed for eligibility, and 11 were excluded due to either including patients with disabilities or not reporting the Frankl scale. Ultimately, eight studies met the inclusion criteria and were incorporated into this review (Figure 1). This systematic approach enhances transparency and methodological rigor in the study selection process.

### 3.1 Study characteristics

The included studies examined the effects of virtual reality (VR) interventions in children aged between 4 and 12 years. Study designs included randomized controlled trials and *in vivo* studies. Subjective outcome measures commonly assessed anxiety and behaviour using scales such as the Modified Children's Dental Anxiety Scale (MCDAS), Corah Dental Anxiety Scale (CDAS), Facial Image Scale, Frankl Behaviour Rating Scale (FBRS), and Venham Image Test. Objective measures included physiological markers such as heart rate, blood pressure, pulse, oxygen saturation, and clinical indicators including the FLACC pain scale, Wong-Baker FACES, and plaque index. Study groups generally compared different VR modalities, conventional distraction techniques (Tell-Show-Do, audio distraction, or mobile games), or alternative non-pharmacological interventions such as Animal-Assisted Therapy (AAT).

Table 1 provides a summary of the study characteristics and Table 2 presents the main study results, with subjective and objective outcomes divided in immersive and non immersive level studies and reported separately to provide a precise understanding of intervention effects. We have also made a Table 3 showing that the majority of the included studies (75%) demonstrated not only statistical significance but also achieved the clinical threshold necessary for procedural success. Specifically, immersive interventions (e.g., Gómez-Polo et al., Pathak et al.) consistently resulted in a shift of at least one point in the Frankl Scale or a > 1 reduction in heart rate, which translates to a tangible improvement in the clinician's ability to perform complex dental treatments safely.

**Table 1:** Study characteristics.

Authors	Year	Age (years)	Study design	Subjective outcome measures	Objective outcome measures	Study groups
Aminabadi et al.	2022	4–6	RCT	MCDAS, FBRS	Plaque Index	Immersive VR, Semi-immersive VR, Non-immersive VR, Control (TSD/audio/no intervention)
Gómez-Polo et al.	2021	5–10	Clinical trial	CDAS, Facial Image Scale, FBRS	–	VR, Control
Pathak et al.	2023	6–12	RCT	Venham Image Test	FLACC, Pain Rating Scale, Wong-Baker FACES, Heart Rate	VR, Control
Balık et al.	2024	7–12	RCT	FBRS	MCDASf	Pre-treatment VR, Control
Jyoti et al.	2024	4–8	Clinical Trial	Behaviour index	Anxiety index	VR, On-screen distraction
Shams et al.	2024	6–10	RCT	–	MVARS	VR, TSD, Audio distraction
Pande et al.	2020	5–8	RCT	Facial Image Scale	Pulse rate, Blood Pressure	VR, TSD, Audio, Mobile games
Kashyap et al.	2025	6–12	RCT	MCD	SEM, VAS, Pulse rate, Oxygen saturation	VR, AAT, Playroom

**Table 2:** Synthesis of results categorized by virtual reality (VR) immersion level.

VR category	Study (year)	Primary outcome (behavior/anxiety)	Subjective results	Objective results	Effect direction
	Gómez-Polo et al. (2021)	Frankl Behavior Scale (FBRS)	95% happy; 100% positive behaviour vs. Control 62% happy; 68% positive behaviour	-	↑
	Pathak et al. (2023)	Heart Rate (HR)	Venham Image Test: VR 2.1 ± 0.8 vs. Control 4.0 ± 1.2	Heart rate: VR 88 ± 6 bpm vs. Control 101 ± 8 bpm; FLACC: VR 1.5 ± 0.6 vs. Control 3.2 ± 0.9	↓
Immersive VR (HMD with total visual occlusion)	Pande et al. (2020)	FBRS/Vital Signs	Facial Image Scale: VR 1.8 ± 0.4 vs. TSD 3.1 ± 0.5	Pulse: VR 82 ± 5 bpm vs. TSD 95 ± 6 bpm; BP: VR 92/56 mmHg vs. TSD 105/64 mmHg	↑/↓
	Shams et al. (2024)	MCDAS(f)/Pulse Rate	.	MVARS: VR 2.2 ± 0.7 vs. TSD 3.6 ± 0.8 vs. Audio 3.4 ± 0.6	↑/↓
	Kashyap et al. (2025)	FLACC/FBRS	MCD: VR 10.4 ± 1.1; AAT 10.6 ± 1.2 vs. Playroom 14.3 ± 1.5	SEM, VAS, Pulse: VR & AAT lower than Playroom; Oxygen saturation stable	↑
	Jyoti et al. (2024)	Venham Picture Test/HR	Behaviour index: VR 4.8 ± 0.3 vs. On-screen 3.5 ± 0.4	Anxiety index: VR 9.6 ± 1.2 vs. On-screen 13.5 ± 1.5	↓
Semi-Immersive/Non-Immersive (VR Glasses with peripheral vision or interactive screens)	Aminabadi et al. (2022)	Children's Distress (Drawing)	MCDAS: VR 10.2 ± 1.1 vs. Control 14.5 ± 1.5; FBRS positive behaviour: 92% vs. 65%	Plaque Index: No significant difference	↓
	Balık et al. (2024)	FIS/Wong-Baker FACES	FBRS: VR 4.6 ± 0.5 vs. Control 3.2 ± 0.6	MCDASf: VR 11.8 ± 2.0 vs. Control 16.4 ± 2.2	↑

**Table 3:** Summary of effect directions and clinical magnitude of VR intervention.

Study (Year)	Primary Outcome Measure	Effect Direction	Effect Magnitude (MD/Range)	Clinical Threshold Achievement^1^
Gómez-Polo et al. (2021)	Frankl Scale (FBRS)	↑	+1.2 pts increase in FBRS	Yes (Significant behavioral shift)
Pathak et al. (2023)	Heart Rate (BPM)	↓	−13 bpm (mean difference)	Yes (>10% reduction in stress)
Pande et al. (2020)	Pulse/Frankl Scale	↑/↓	Significant BP & Pulse drop	Yes (Improved physiological stability)
Shams et al. (2024)	MCDAS(f)/Pulse	↑/↓	MD: −4.2 in anxiety scores	Yes (Transition to “low anxiety”)
Kashyap et al. (2025)	FLACC (Pain)	↑	35% reduction in pain scores	Yes (Enhanced procedural comfort)
Jyoti et al. (2024)	Venham/Heart Rate	↓	−8 to −12 bpm during LA^2^	Yes (Mitigated peak stress)
Aminabadi et al. (2022)	Distress (Drawing)	↓	Significant score reduction	Partial (Subjective projective shift)
Balık et al. (2024)	FIS/FACES Scale	↑	Moderate anxiety decrease	Partial (Effective for mild cases)

Legend: ↑ Superiority over control (Behavior/Cooperation); ↓ Superiority over control (Anxiety/Stress reduction); ↔ Equal to control. ^1^Clinical Threshold: Defined as a shift of ≥1 point in Frank Scale or >10% reduction in physiological stress markers.

2 LA: Local Anesthesia.

### 3.2 Data synthesis

Due to heterogeneity in interventions, outcome measures, and patient populations, a quantitative meta-analysis was not feasible. Subjective outcomes consistently indicated that VR significantly reduced anxiety and improved cooperative behaviour in paediatric dental patients. Immersive VR was associated with lower MCDAS/F scores and higher positive behaviour ratings, with statistically significant differences compared to controls. Gómez-Polo et al. (2021) reported that 95% of children using VR were during treatment compared with 62% in the control group, demonstrating a clinically meaningful improvement (12).

Behavioural indices across multiple studies (2, 13) also reflected enhanced compliance and cooperation with VR interventions.

Objective outcomes complemented these findings, showing that VR mitigated physiological stress responses. Pathak et al. (2023) (14) demonstrated lower heart rates during dental procedures (VR 88 ± 6 bpm vs. Control 101 ± 8 bpm), and Pande et al. (2020) (15) reported reductions in pulse and blood pressure with VR compared to other distraction techniques.

Pain and distress measures such as FLACC and Wong-Baker FACES were also significantly lower in VR groups, indicating reduced procedural discomfort. In addition, VR and Animal- Assisted Therapy were effective in maintaining stable oxygen saturation while reducing pulse and anxiety scores relative to conventional playroom activities (16). Across studies, the magnitude of the effect was largest for immersive VR, with consistent statistical significance for both subjective and objective measures.

### 3.3 Quality assessment

Risk of bias was assessed using the RoB 2 tool (Table 4). High-risk concerns were primarily associated with outcome measurement, due to lack of blinding and reliance on subjective behavioural scales. Several studies also lacked prospective trial registration or pre-specified analysis plans. While some trials (15–17) demonstrated methodological rigor and objective outcome assessment, most trials presented high risk of bias in at least one domain. Of the 8 studies included, 5 (62.5%) were classified as having “High Risk” of overall bias, mainly due to issues in the “Outcome Measurement” domain. The remaining 3 (37.5%) were classified as having 'Some Concerns'. None of the included studies fell into the “Low Risk” category.

**Table 4:** Risk of bias assessment (RoB 2 tool).

Authors	Randomization	Deviations	Missing data	Outcome measurement	Selective reporting	Overall bias
Jyoti et al. 2024	?	+	+	-	?	-
Shams et al. 2024	?	+	+	-	?	-
Pande et al., 2020	+	+	+	+	?	?
Gómez-Polo et al. 2021	+	+	+	-	?	-
Aminabadi et al. 2022	+	+	+	?	+	?
Pathak et al. 2023	?	+	+	-	?	-
Balık and Usluoğlu 2024	+	+	+	-	?	-
Kashyap et al. 2025	+	+	+	+	+	+

* + = Low risk;? = Some concerns; - = High risk.

### 3.4 Quantitative synthesis

The certainty of evidence was evaluated using the GRADE approach further refined with domain-specific criteria relevant to pediatric dentistry expressed in Tables 5, 6 (10).

**Table 5:** Evidence quality assessment (GRADE).

Authors	Overall effect	Grade	Justification
Jyoti et al. 2024	VR superior to on-screen VR superior to on-screen distraction	Moderate	Methodological limitations (no blinding) but large, consistent effect
Shams et al. 2024	VR favourable trend, not significant	Low	Small sample size, non-significant intergroup differences
Pande et al. 2020	VR reduced anxiety & physiological stress	Moderate	Statistically significant, consistent results; limited sample size
Gómez-Polo et al. 2021	VR clearly superior (95% happy, 100% positive behaviour)	High	Robust methodology, large sample, significant results
Aminabadi et al.al. 2022	VR superior in reducing anxiety & enhancing compliance	Moderate	Strong findings; single-centre, unclear blinding limits certainty
Pathak et al. 2023	VR beneficial for physiological anxiety control	Low	Small sample, lack of blinding, significance limited to physiological measures
Balık and Usluoğlu, 2024	VR effective for anxiety reduction & cooperation	High	Well-designed RCT, adequate sample, robust improvements
Kashyap et al. 2025	Both AAT & VR reduced anxiety & improved behaviour	Moderate	RCT with transparent methodology; downgraded for single-centre, limited sample, no long-term follow-up

**Table 6:** Evidence Quality Assessment (GRADE) with domain-specific details.

Outcome	Anxiety reduction (Frankl scale)	Physiological stress (heart rate)	Pain perception (Flacc/Won-Bkaer)	Cooperative behavior (SEM scale)
N° of studies	8	6	4	3
Risk of bias	Serious	Not serious	Serious	Serious
Inconsistency	Not serious	Not serious	Serious	Not serious
Indirectness	Serious	Not serious	Not serious	Not serious
Imprecision	Serious	Not serious	Serious	Serious
Certainty	Low	High	Very low	Moderate
Domain-Specific reasons of downgrading (Pediatric Dentistry)	Lack of blinding in the dental chair (observer bias). Heterogeneity in dental procedures (Prophylaxis vs. extractions). Small sample sizes and lack of baseline anxiety screening	Objective telemetry (BPM) provides high certainty as it is less prone to subjective observer bias of parental interference during treatment	High variability in individual pain thresholds and potential “novelty effect” bias where technology temporarily masks procedural discomfort	Performance bias due to the dentist´s own technology acceptance” profile, which can indirectly influence the child´s clam

Two studies (12, 18) were graded high certainty, reflecting robust methodology, adequate sample sizes, and statistically significant outcomes. Four studies (13, 15–17) achieved moderate certainty due to small sample sizes or single-centre designs, despite consistent findings favouring VR.

Two studies (14, 19) were classified as low certainty, primarily because of very small samples, lack of blinding, and significance limited to physiological measures. Overall, the body of evidence indicates that VR interventions are effective in reducing anxiety, improving cooperative behaviour, and mitigating physiological stress responses in paediatric dental patients, although further rigorously designed studies are warranted to strengthen certainty.

Beyond standard parameters, the assessment accounted for the “novelty effect” inherent in digital health interventions and the procedural heterogeneity (ranging from non-invasive hygiene to surgical extractions). A significant downgrade in certainty for behavioral outcomes (Frankl and SEM scales was applied due to the impossibility of blinding the operator and patient in the dental chair, which introduces a high risk of observer bias. Conversely, objective physiological markers, such as heart rate and oxygen saturation, maintained a higher level of certainty as they provide a direct, automated measure of the autonomic nervous system's response to dental stress, independent of the clinician's interpretation.

Furthermore, following recent guidelines on dental digital literacy (20), the evidence was penalized for “indirectness” in studies that failed to stratify participants by baseline dental anxiety, a the therapeutic magnitude of VR is known to vary significantly between cooperative and phobic patients.

## 4 Discussion

The findings of this systematic review demonstrate that virtual reality (VR) is an effective tool for reducing anxiety and enhancing behaviour in paediatric dental patients (12–19) Across the included studies, immersive VR consistently produced the strongest effects, suggesting that the level of sensory engagement plays a critical role in patient response. Immersive VR, by fully engaging visual and auditory attention, appears to divert children's focus from potentially stressful dental stimuli, thereby decreasing anxiety and facilitating cooperation (17, 19). Semi-immersive and non-immersive VR provided intermediate benefits, while conventional techniques such as Tell-Show-Do (TSD) were comparatively less effective (17).

These results align with theories of attention distraction and cognitive load, which posit that more engaging and immersive experiences can reduce the cognitive and emotional resources available to process stressors, thereby diminishing anxiety and behavioural resistance.

A critical appraisal of the included studies reveals that 62.5% are at a high risk of bias, particularly within the outcome measurement domain. In the context of paediatric dentistry, the impossibility of blinding the operator and the patient to the Virtual Reality (VR) intervention creates a significant performance and detection bias that must be addressed. This methodological frailty likely leads to an overestimation of the effect size regarding cooperative behaviour; clinicians who are aware of the intervention may subconsciously score the child's compliance more favourably on the Frankl Scale.

Furthermore, the ‘novelty effect'—the immediate positive reaction to a new digital stimulus—may inflate short-term success rates, masking the true baseline anxiety. Therefore, while the statistical significance across studies is consistent, the perceived clinical magnitude of VR’s effectiveness should be interpreted with caution, as it may be partially amplified by the lack of rigorous double-blinding and the subjective nature of traditional behavioural rating scales.

The effectiveness of VR was evident across both subjective and objective measures. Behavioural outcomes assessed using the Frankl Behaviour Rating Scale revealed that children exposed to immersive VR demonstrated the highest levels of positive behaviour, whereas semi-immersive and non-immersive VR groups showed moderate improvement (12, 13, 17). The pattern observed suggests that immersive experiences are particularly beneficial in maintaining attention and minimizing disruptive behaviours. These improvements were corroborated by other validated instruments, such as the Sound–Eye–Motor (SEM) scale and the Visual Analogue Scale (VAS), which reflected reductions in anxiety and greater cooperative engagement during dental procedures (16). Even in studies where post-treatment Frankl scores were not recorded, physiological markers such as heart rate and pulse consistently demonstrated decreased stress responses in VR groups (14, 15), supporting the notion that VR exerts both behavioural and autonomic effects.

The benefits of VR extended to both conservative and surgical dental treatments. In non- invasive procedures such as restorations and prophylaxis, VR was shown to reduce preoperative anxiety and improve cooperation, especially when applied prior to treatment, indicating that preparatory exposure can optimize the child's behavioural response (12, 13, 18). In the context of surgical interventions, such as primary molar extractions, VR effectively lowered heart rates and other physiological indicators of stress, suggesting that distraction alone can mitigate anticipatory and procedural anxiety (14, 15). Audiovisual VR interventions, in particular, appeared most effective in addressing both physiological and behavioural dimensions of fear in uncooperative children (15).

Comparisons with other non-pharmacological interventions reinforce the relative superiority of VR. Screen-based or auditory distractions provided some benefit but were generally less engaging, resulting in smaller reductions in anxiety and less consistent improvements in cooperation (13, 19). Similarly, while Animal-Assisted Therapy (AAT) showed comparable benefits in reducing anxiety and enhancing cooperation in controlled settings, VR offered a more controlled, repeatable, and widely applicable intervention that can be standardized across dental clinics (16). These findings suggest that VR can be effectively integrated as an adjunct to conventional behaviour management strategies, complementing methods such as TSD rather than replacing them (12, 17).

The observed improvements in behaviour and anxiety management can be explained by the immersive, multi-sensory nature of VR. By engaging the child's visual and auditory attention, VR diverts cognitive focus from the dental procedure, reducing perception of discomfort and anxiety (13, 19). This is consistent with attentional and cognitive theories of distraction, which posit that competing sensory stimuli can lower the subjective experience of stress and pain.

Moreover, VR can create an enjoyable and interactive environment, which may enhance the child's sense of control and predictability during the procedure, further promoting cooperative behaviour (17, 18).

Despite these promising findings, certain limitations must be acknowledged. Variations in VR modalities, study designs, outcome measures, and patient ages introduce heterogeneity that complicates direct comparisons (15, 16). Some studies relied on unblinded behavioural assessments, which may have introduced observer bias (12). Small sample sizes in several trials limit generalizability, and long-term effects of VR interventions remain largely unexplored (14).

Nevertheless, the convergence of results across multiple studies supports the conclusion that VR is a valuable, safe, and effective tool for managing anxiety and behaviour in paediatric dental settings (17, 19).

As recent research in the field of dental education suggests (20), the potential of VR goes beyond mere distraction. The key mechanism lies in these platforms' ability to offer “behavioural modelling” by allowing children to explore the clinical environment in a playful way before facing the actual procedure. This reduces fear of the unknown through an immersive educational experience that the traditional Tell-Show-Do method cannot replicate with the same sensory intensity.

In summary, immersive VR consistently emerges as the most effective non-pharmacological intervention for reducing anxiety and improving cooperative behaviour in children during dental procedures (12, 15, 17). Semi-immersive and non-immersive VR offer moderate benefits, while conventional techniques provide comparatively limited improvements. The efficacy of VR is supported by both subjective measures of anxiety and behaviour and objective physiological markers, highlighting its multifaceted impact. These findings suggest that VR can be implemented as a routine adjunct in paediatric dentistry to enhance patient experience and facilitate treatment, particularly for children who exhibit higher levels of dental anxiety or behavioural challenges (16).

## 5 Limitations

In conducting our systematic review we identified some methodological limitations consistent with those highlighted by Alonso-Coello et al. in 2016 (21). We reported most outcomes in relative or descriptive terms, such as percentage reductions in anxiety or improvements in cooperative behaviour, without presenting absolute effect estimates like absolute risk reduction or number needed to treat. We also recognise that baseline risks and absolute event rates for anxiety or behavioural deterioration were not specified, which constrains the capacity to determine the true magnitude of benefit across populations.

Additionally, our synthesis was affected by heterogeneity and imprecision, as the included studies employed different behavioural and anxiety assessment tools (Frankl, CFSS-DS, VPT, etc.) and diverse VR modalities (immersive and semi-immersive). We acknowledge that expressing results solely in relative terms may overstate the intervention's benefit. These limitations mirror those described in the wider literature and underline the necessity of reporting absolute effects and baseline risks to enhance transparency, interpretability, and clinical applicability in future systematic reviews.

This systematic review indicates that virtual reality (VR) is an effective non-pharmacological tool for managing anxiety and improving cooperative behaviour in paediatric dental patients.

Across a range of procedures, including oral hygiene instruction, restorative treatments, and tooth extractions, both immersive and semi-immersive VR systems, such as headsets and VR glasses, consistently demonstrated beneficial effects. Behavioural outcomes, measured using instruments like the Frankl Behaviour Rating Scale, and anxiety outcomes, assessed through validated scales and physiological indicators such as heart rate, uniformly showed that VR interventions reduced distress and enhanced cooperation. The level of immersion appeared to influence effectiveness, with immersive VR producing the most pronounced improvements.

Overall, we can conclude that evidence supports integrating VR as an adjunct to conventional behaviour management strategies, providing children with a more engaging, controlled, and reassuring clinical experience while facilitating smoother and safer dental care.

Furthermore, this review identifies a potential novelty bias; the reported efficacy may be linked to the novelty of the experience for paediatric patients, and its long-term sustainability in recurrent treatments remains uncertain. Similarly, we cannot rule out publication bias, whereby null or negative results from technological interventions tend to be under-represented in the current literature. Finally, the lack of standardisation in the software used introduces a content bias that makes it difficult to discern whether the benefit stems from visual immersion or from the specific quality of the recreational material employed.

Why this paper is important to paediatric dentists and paediatricians:

1. Demonstrates clinical efficacy, showing that immersive VR effectively reduces dental anxiety and improves cooperation in children, supported by behavioural and physiological measures.
2. Guides modality choice: Identifies immersive audiovisual VR as the most effective compared to semi-immersive, non-immersive, and traditional distraction techniques.
3. Highlights research limitations: documents methodological variation and bias risk, stressing the need for larger, standardized, well-designed RCTs before wider clinical use.

To make progress in this field, and in line with the recent recommendations of the ADA (2025) (22), future research must move away from single-centre pilot studies. Priority should be given to: (a) the use of blinded assessors via video analysis, (b) the monitoring of Heart Rate Variability (HRV) as a real-time marker of anxiety, and (c) the evaluation of the impact of VR on the learning curve of the novice paediatric dentist. Only through longitudinal data will we be able to determine whether VR is a tool for lasting desensitisation or a fleeting distraction.

## Data Availability

The raw data supporting the conclusions of this article will be made available by the authors, without undue reservation.
